# Structural basis for Klf4 recognition of methylated DNA

**DOI:** 10.1093/nar/gku134

**Published:** 2014-02-11

**Authors:** Yiwei Liu, Yusuf Olatunde Olanrewaju, Yu Zheng, Hideharu Hashimoto, Robert M. Blumenthal, Xing Zhang, Xiaodong Cheng

**Affiliations:** ^1^Department of Biochemistry, Emory University School of Medicine, Atlanta, GA 30322, USA, ^2^New England Biolabs, 240 County Road, Ipswich, MA 01938, USA and ^3^Department of Medical Microbiology and Immunology and Program in Bioinformatics, The University of Toledo College of Medicine and Life Sciences, Toledo, OH 43614, USA

## Abstract

Transcription factor Krüppel-like factor 4 (Klf4), one of the factors directing cellular reprogramming, recognizes the CpG dinucleotide (whether methylated or unmodified) within a specific G/C-rich sequence. The binding affinity of the mouse Klf4 DNA-binding domain for methylated DNA is only slightly stronger than that for an unmodified oligonucleotide. The structure of the C-terminal three Krüppel-like zinc fingers (ZnFs) of mouse Klf4, in complex with fully methylated DNA, was determined at 1.85 Å resolution. An arginine and a glutamate interact with the methyl group. By comparison with two other recently characterized structures of ZnF protein complexes with methylated DNA, we propose a common principle of recognition of methylated CpG by C2H2 ZnF proteins, which involves a spatially conserved Arg–Glu pair.

## INTRODUCTION

The control of gene expression in mammals relies in part on the modification status of DNA cytosine residues, which exist in at least five forms: cytosine (C), 5-methylcytosine (5mC), 5-hydroxymethylcytosine (5hmC), 5-formylcytosine (5fC) and 5-carboxylcytosine (5caC) (1–4). DNA methyltransferases methylate cytosines in the context (primarily) of CpG dinucleotides, generating 5mC in the genome (5,6). Ten-eleven translocation (Tet) dioxygenases convert 5mC to 5hmC, 5fC and 5caC in three consecutive oxidation reactions (7–10). The exact functions of these oxidized cytosine bases are under investigation.

The cytosine modifications can be ‘interpreted’ or ‘read’ by effector (or reader) molecules. There are currently three best-known classes of mammalian proteins containing domains that recognize modified DNA. The first class includes methyl-binding domains that recognize methylated cytosine in fully methylated CpG dinucleotides (11). The second class includes Su(var)3-9, En(zeste), and Trithorax (SET) and really interesting new gene (RING) finger-associated domains that recognize hemimethylated CpG sites—transiently generated during DNA replication and methylated on the parental strand only (12). The third class of mammalian proteins that recognize methylated DNA is the C2H2 zinc finger (ZnF) proteins that preferentially bind to methylated CpG within a longer specific DNA sequence (13). This unique feature of ZnF proteins is important in that ‘sequences longer than CpG would be necessary for the regulation of gene expression by methylation’ (14).

Recently, ZnF DNA-binding domains from two proteins, Kaiso and Zfp57, were structurally analyzed in complex with their respective methylated DNA elements (15,16). Here we analyze the interaction of transcription factor Krüppel-like factor 4 (Klf4) with its target methylated DNA element. By comparing three examples of ZnF-methylated DNA interactions, we have derived an apparent consensus sequence motif associated with recognition of methylated CpG elements.

**Figure 1.**
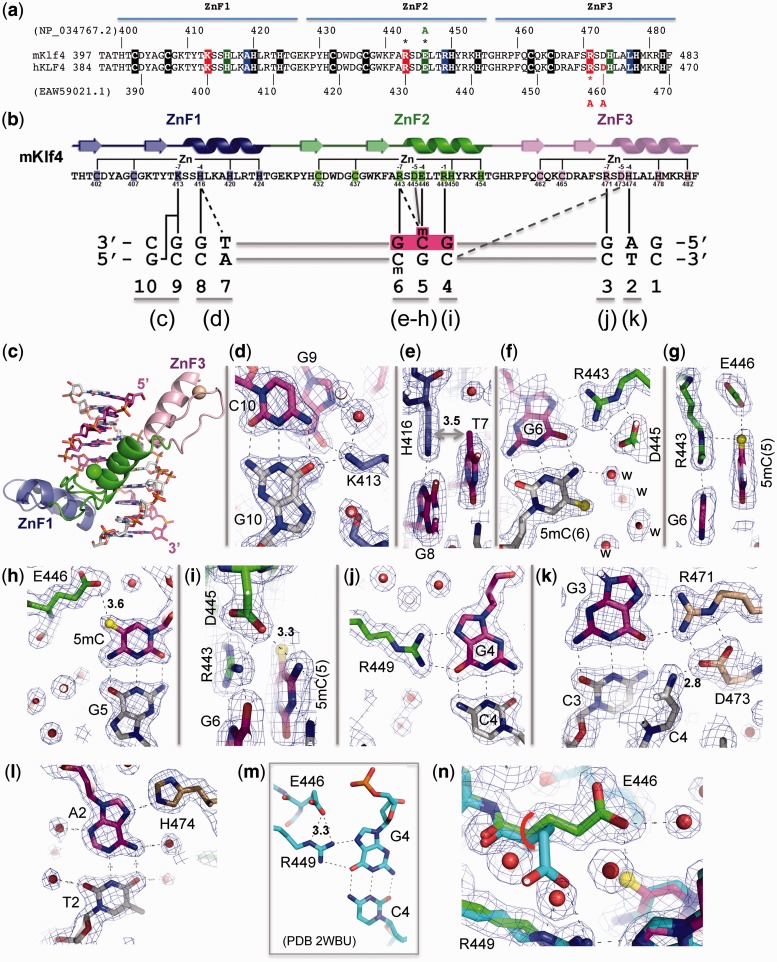
Klf4 binds methylated CpG. (**a**) Sequence alignment of the C-terminal ZnF DNA-binding domains of mouse Klf4 (mKlf4) and human KLF4 (hKLF4), which are identical in sequence. The mutations made by Hu *et al.* (22), R458A and D460A of hKLF4 are located in the last (third) ZnF, which does not directly participate in methyl-CpG binding. (**b**) Schematic representation of mKlf4 DNA-binding ZnF domain. The sequence and the secondary structure are shown as follows: (arrows) β strands and (ribbons) α helices. The positions highlighted are responsible for Zn ligand binding (C2H2) and DNA base-specific interactions at −1, −4, −5 and −7 positions (relative to the first zinc-binding histidine): solid lines (direct hydrogen bonds) and dashed lines (van der Waals contacts). The DNA sequence used for the study is shown with the majority of base interactions involving the top strand from 3′-to-5′ (left-to-right). The central GCG sequence is colored in magenta and the letter ‘m’ indicates the methyl group in 5mC. Dotted and solid vertical lines indicate specific binding interactions. (**c**) The mKlf4 ZnF protein binds in the major groove of DNA with ZnF1 (blue), ZnF2 (green) and ZnF3 (pink). (**d**) Lys413 of ZnF1 at the −7 position interacts with the O6 oxygen atoms of both guanines at G9 (of upper strand) and G10 (of lower strand). (**e**) His416 of ZnF1 at the −4 position interacts with the TpG dinucleotide. (**f**) Arg443–Gua6 interaction; a layer of ordered water molecules (marked ‘w’) shields the methyl group of lower strand 5mC. (**g**) The upper strand 5mCpG interacts with Arg443 and forms a 5mC-Arg-Gua triad. (**h**) One of the carboxylate oxygen atoms of Glu446 forms a weak C-H…O type of hydrogen bond with the methyl group of the upper strand 5mC. (**i**) Asp445 of ZnF2 at the −5 position interacts with Arg443 at the −7 position and the N4 atom of 5mC of the upper strand. (**j**) Arg449–G4 interaction. (**k**) Arg471–G3 interaction; Asp473 of ZnF3 at the −5 position interacts with Arg471 at the −7 position and the N4 atom of Cyt4 of the lower strand. (**l**) His474–A2 interaction. (**m** and **n**) Structural comparison of mKlf4 Glu446 in the absence (m) and presence of methylation (n).

Klf4 is one of 26 members of the specificity protein/Krüppel-like factor (Sp/Klf) family of ZnF transcription factors (17–19) and is one of the four Yamanaka reprogramming factors (20). Two recent studies suggested Klf4 binds specific methylated and/or unmethylated elements. Using a DNA pull-down approach combined with quantitative mass spectrometry, three Klf proteins (Klf2, Klf4 and Klf5) were identified as 5mC readers in mouse embryonic stem (ES) cells (21). Using a protein microarray-based approach, 47 human transcription factors including human KLF4 could bind to methylated CpG sites (22). Both mouse Klf4 and human KLF4 proteins share an identical DNA-binding domain composed of three standard Krüppel-like ZnFs (Figure 1a). The consensus-binding element for Klf4 was determined by both base-specific mutagenesis [5′-(A/G)(G/A)GG**(C/T)G**(C/T)-3′] (18) and chromatin immunoprecipitation sequencing (ChIP-seq) [5′-GGG**(C/T)G**(T/G)GG-3′] (23). These both share a central GG(C/T)G, which contains either CpG, which can be methylated, or TpG, which is intrinsically methylated on one strand and can be methylated on the other strand (CpA) by DNA methyltransferase 3a (Dnmt3a) (24,25). In other words, as with Kaiso (see below), TpG can substitute for the (±methyl)-CpG dinucleotide in the consensus sequences, and has a methyl group in the same position as methylated C (5-carbon of the pyrimidine). Here we analyze the Klf4 interactions with methylated DNA both structurally and biochemically.

## MATERIALS AND METHODS

### Protein expression and purification

For mouse Klf4, Glutathione S-transferase (GST)-tagged Klf4 fragment (residues 396–483; pXC1248) and its mutant Glu446-to-alanine (E446A; pXC1257) were cloned into pGEX6P-1 vector and expressed in *Escherichia coli* BL21-CodonPlus(DE3)-RIL (Stratagene). Bacterial cells were cultured at 37°C in the Luria–Bertani medium and induced for protein expression with 0.2 mM isopropyl β-D-1-thiogalactopyranoside at 16°C overnight. The bacteria were harvested and lysed by sonication in 20 mM Tris–HCl (pH 7.5), 250 mM NaCl, 5% (v/v) glycerol and 0.5 mM tris(2-carboxyethyl)phosphine (TCEP), followed by centrifugation for 35 min at 18 000 rpm (SA-300 rotor). After purification on Glutathione Sepharose 4B (GE Healthcare), the GST tag on the recombinant protein was removed by PreScission protease (purified in-house), resulting in the additional N-terminal residues Gly-Pro-Leu-Gly-Ser (GPLGS) relative to the wild type (WT) sequence. Protein was further purified on HiTrap-Q, HiTrap-SP and Superdex-200 (16/60) (GE Healthcare) and concentrated to ∼20 mg ml^−^^1^ in 20 mM Tris–HCl (pH 7.5), 200 mM NaCl, 5% (v/v) glycerol and 0.5 mM TCEP. The yield of the mutant E446A protein was ∼10% that of the wild-type protein.

### Crystallography

The purified Klf4 protein was incubated with annealed oligonucleotides at an equimolar ratio for 0.5 h on ice before crystallization. The final solution contained 0.8 mM protein–DNA complex. Crystals were obtained by the sitting-drop method; the mother liquor contained 100 mM Tris–HCl (pH 8.5), 250 mM NaCl and 20% polyethylene glycol 8000. Crystals grew within 3 days at 16°C.

The crystals were flash frozen by plunging into liquid nitrogen. X-ray diffraction data were collected at the SER-CAT beamline at the Advanced Photon Source, Argonne National Laboratory. HKL2000 (26) and CCP4 packages (27) were used for the data processing. The structure was solved by molecular replacement with the coordinates of 2WBU (28) as an initial searching model using the PHENIX (29) and Phaser programs (30). Model refinement was performed with COOT (31) and PHENIX. Molecular graphics were generated with the Pymol program (DeLano Scientific LLC). The Dali server (32) was used for determining the root-mean-squared deviations of different structures.

### DNA-binding assay by fluorescence polarization

**Figure 2.**
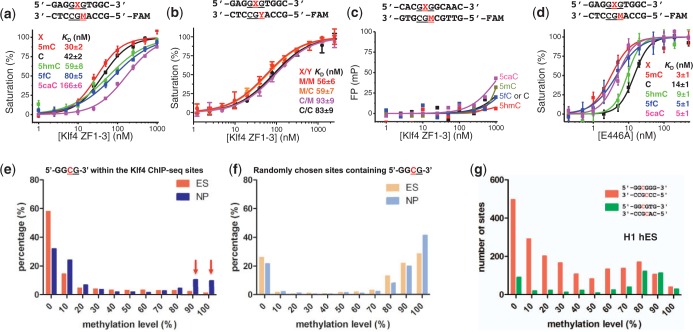
The effects of CpG modifications and DNA sequence on DNA-binding by Klf4. (**a**) Binding affinities measured by fluorescence polarization assays between Klf4 and DNA with five different modification states on the upper strand (5mC = M, C, 5hmC, 5fC and 5caC). (**b**) Binding affinities measured between Klf4 and DNA that is fully methylated, unmodified or hemimethylated (on either strand). For these experiments, only M (5mC) and C were used. (**c**) A GCG-containing DNA sequence partially matching the consensus binding element of Klf4 (underlined) was used as a negative control. Fluorescence polarization (FP) is measured in millipolarization (mP). (**d**) Binding affinities measured between the E446A variant of Klf4 and DNA having five different modification states on the top strand. In all cases, the lower strand has M (5mC). (**e**) Distribution of DNA CpG methylation in mouse ES cells and NP cells (33) that is present within the core GGCG Klf4-ChIP sites identified in ES cells (23). The red arrows indicate changes of methylation levels, from hypomethylation in mouse ES cells to hypermethylation in NP cells (see Table 2), during differentiation and/or reprogramming. (**f**) Distribution of DNA methylation of randomly chosen GGCG sites in the mouse ES genome. (**g**) Distribution of DNA methylation in human H1 ES cells presented within the human KLF4-ChIP sites (34). GGCGTG sequences (green) have a higher proportion of methylated sites than do GGCGGG sequences.

Fluorescence polarization assays for Klf4 DNA binding were performed in 20 mM Tris–HCl (pH 7.5), 150 mM NaCl, 5% (v/v) glycerol and 0.5 mM TCEP at room temperature (∼22°C) using a Synergy 4 Microplate Reader (BioTek). Fluorescently labeled double-stranded DNA probe (10 nM for WT or 1 nM for the E446A mutant) and various amounts of Klf4 protein, with a final volume of 50 µl, were incubated in a 384-well plate for 0.5 h before measurement. The sequences of 6-carboxy-fluorescein (FAM)-labeled double-stranded oligonucleotides were FAM-5′-TT GCC AYG CCT C-3′ and 3′-CGG TGX GGA G-5′ (where Y = C or 5mC, and X = C, 5mC, 5hmC, 5fC or 5caC). The control DNA sequences were FAM-5′-GTT GCM GCG TG-3′ and 3′-CAA CGG XGC AC-5′. Curves were fit individually using Origin 7.5 software (OriginLab). *K*_D_ values were calculated as [mP] = [maximum mP] × [C]/(KD + [C]) + [baseline mP], where [mP] is millipolarization and [C] is protein concentration. Averaged *K*_D_ and its standard error were reported. We have found that the absolute magnitude of binding affinity by Klf4 is sensitive to the percentage of glycerol used in the reaction; therefore, absolute (not relative) *K*_D_ values vary somewhat among experiments done at different times (Figure 2a and b).

### Genomic analysis of methylation profiles within Klf4-binding sites

For the methylation profile in the mouse Klf4-binding sites in ES cells, the published Klf4 ChIP-seq (Gene Expression Omnibus (GEO) data set GSM288354) (23) and methylome data in both ES cells and neuronal progenitor (NP) cells (GEO data set GSE30202) (33) were used. We recorded the frequency of the methylation levels at the GGCG sites in the 60-nt windows centered around the midpoints of the reported ChIP-seq peak regions (Figure 2e). Of the 10 297 Klf4 ChIP-seq peak regions in the mouse ES cells, 5530 contain the GGCG motif. As a comparison, we randomly picked an equal number of 60-nt windows in the mouse ES genome and carried out the same analysis (Figure 2f). Similarly, for the methylation profiles in Klf4-binding sites in human H1 cells, the published KLF4 ChIP-seq (GEO data set GSM447584) (34) and methylome data (GEO data set GSM432685) (34) were used. Mouse genome mm9 and human genome hg19 were used in these analyses.

## RESULTS AND DISCUSSION

### Structure of Klf4 bound with methylated DNA

Klf4 contains three tandem C2H2 fingers at its C-terminus (Figure 1a). A structure was previously determined for mouse Klf4 ZnF domain bound to the 10-bp oligonucleotide (5′-GAGG**CG**TGGC-3′) (28), which is present in the basic transcriptional element of *CYP1A1* gene (18). We modified this 10-bp DNA to contain a fully methylated CpG site and determined the complex structure of Klf4 at a resolution of 1.85 Å (Table 1). Except for the side chain of Glu446 (see below), the overall structure of the Klf4 is essentially unchanged between complexes with methylated or unmodified DNA, with a root-mean-squared deviation of <0.5 Å when comparing 85 pairs of Cα atoms. The three ZnFs of Klf4 bind in the major groove of the DNA (Figure 1b and c). ZnF3 interacts with the 5′ sequence (GAG), ZnF2 interacts with the central (potentially)-methylated GCG and ZnF1 interacts with the 3′ sequence (TGGC) (Figure 1b and c; note, from left to right, the protein sequence runs from N to C termini, whereas the DNA sequence of the recognition strand runs from 3′ to 5′).

**Table 1.** X-ray data collection and refinement statistics

Protein	Klf4
DNA (M = 5mC)	3′-CGGTGMGGAG-5′
	5′-GCCAMGCCTC-3′
Beamline	APS 22-BM
Wavelength (Å)	1.000
Total number of images	360 (1° rotation and 3 s exposure)
Space group	P4_3_2_1_2
Cell dimensions	
* a, b, c* (Å)	48.705, 48.705, 131.015
* α, β, γ* (°)	90, 90, 90
Resolution (Å)^a^	35–1.85 (1.92–1.85)
R_merge_ (%)	0.076 (0.501)
<I >/σ(I)	27.5 (1.8)
Completeness (%)^a^	97.7 (80.4)
Redundancy^a^	12.0 (3.9)
Observed reflections	166 500
Unique reflections^a^	13 918 (1103)
Refinement	
Resolution (Å)	34.44–1.85
Number of reflections	13 843
R_work_ / R_free_ (%)	18.68 / 23.39
Number of atoms	
Protein	694
DNA	438
Water	134
Others	13 (3 Zn^2+^, 1 acetate molecule, 1 glycerol molecule)
B-factors (Å^2^)	21.9 (overall)
Protein	21.5
DNA	20.1
Water	29.4
Others	30.6
Root mean squared deviations	
Bond lengths (Å)	0.006
Bond angle (°)	1.313

^a^Data for the highest-resolution shell are given in parentheses.

As with the Zfp57–DNA complex (16), the two 5mCs of the two DNA strands exhibit different patterns of interaction with Klf4. A layer of ordered water molecules (marked ‘w’ in Figure 1f) envelops the methyl group of 5mC on the bottom strand. In contrast, the methyl group of 5mC in the top strand makes van der Waals contacts with the guanidine group of Arg443, which in turn forms bifurcated hydrogen bonds with the 3′ guanine G6 (Figure 1g), forming a 5mC-Arg-Gua triad (35). In addition, the 5mC methyl group interacts with the carboxylate group of Glu446, forming a weak (3.6 Å) C-H…O type of hydrogen bond (Figure 1h)—a common but underappreciated interaction in biomolecules and molecular recognition (36).

Among the side chains involved in DNA base-specific interactions, Glu446 of Klf4 undergoes one of the largest conformational changes on binding methylated versus unmethylated CpG DNA. In the structure of the Klf4 bound with unmodified DNA (28), the carboxylate group of Glu446 points away from C5 position of the cytosine, and forms a weak hydrogen bond with Arg449, which in turn interacts with the 5′ guanine G4 (Figure 1m). Superimposing the two structures reveals that Glu446 moves from the Arg449-interacting conformation to the 5mC-interacting conformation via a ∼100° rotation of the side chain torsion angle χ1 (Figure 1n).

### Sequence and methyl-specific binding in solution

To verify the structural observation of Klf4 binding to methylated GCG, we used fluorescence polarization analysis to measure the dissociation constants (*K*_D_) between Klf4 fingers and double-stranded oligonucleotides containing a single CpG dinucleotide. Because contact with the 5mC methyl group on the bottom strand involves only water-mediated interactions (Figure 1f), we initially altered only the top strand, replacing the 5mC with unmodified cytosine (C) or three different oxidative modifications (5hmC, 5fC and 5caC), with 5mC on the bottom strand in all cases. The binding affinity for fully methylated (M/M) DNA is slightly stronger than that of hemimethylated (C/M) DNA under the assay conditions (Figure 2a), although the difference is only ∼40%. Each oxidation event, from 5mC to 5hmC to 5fC to 5caC, resulted in progressively weaker binding (by factors of ∼2, 3 and 6, respectively). We repeated these experiments with the same oligonucleotides either unmodified (C/C), hemimethylated on one strand (M/C or C/M) or fully methylated (M/M) (Figure 2b). Klf4 shows similar affinity for DNA with fully methylated (M/M) and hemimethylated on the top strand (M/C), with slightly reduced affinity by a factor of ∼1.5 for unmodified DNA (C/C) and hemimethylated DNA on the bottom strand (C/M).

Thus, methylation had significant, though modest, effects on binding. In contrast, mutating the sequence outside of the central GCG abolished Klf4 binding, regardless of the GCG modification status (Figure 2c). Together, these data indicate that the interaction between Klf4 and DNA depends largely on the specific sequence context and significantly but less profoundly on the cytosine modification state. This observation differs from that of Sprujit *et al.* who used a DNA pull-down with recombinant GST-Klf4-ZF domain, followed by western blotting against GST (21). Two examples with different DNA sequences were shown with a sequence containing four repeats of GAC and a sequence containing three overlapping Klf4 consensus motifs. In both instances, western blots indicated the highest binding to oligonucleotides containing 5mC (21), even though the (GAC)_4_ sequence does not resemble the Klf4 consensus sequence.

The structural results strongly implicate Glu446 in cytosine modification discrimination, so we replaced the negatively charged Glu446 of mouse Klf4 with alanine (E446A). The E446A mutant exhibited no detectable selectivity of methylated over oxidative derivatives (5fC and 5caC), though it maintained 5mC selectivity over unmodified and 5-hydroxymethylated cytosines (C and 5hmC) (Figure 2d). This change in selectivity is not via decreased relative affinity for 5mC, but rather via an increase in the relative affinities for 5fC and 5caC. Like the corresponding glutamate residue in Zfp57 (37), the side chain of Glu446 in Klf4 (the size and the charge) is dispensable for methyl group recognition. Extensive substitution study of the corresponding glutamate in Zfp57 suggested that the negatively charged glutamate side chain carboxylate group might be critical in discriminating against the negatively charged carboxylate moiety of 5caC (37).

Using previously published data sets of Klf4 ChIP-seq profiles (23) and bisulphite-sequenced methylomes (33), we then examined the methylation status of the CpG site in the core Klf4 binding motif GGCG. Our analyses indicate that a substantial number of Klf4-binding sites in mouse (Figure 2e) and human ES cells (Figure 2g) are methylated (21). Importantly, many of the unmethylated Klf4-binding sites in mouse ES cells become hypermethylated in NP cells (33) (Figure 2e). Table 2 lists 15 such sites containing the 5′-GGCGTG-3′ Klf4-binding sequence that exhibit both hypomethylation (<20%) in ES cells and hypermethylation (>80%) in NP cells. This suggests that Klf4 may bind methylated loci in differentiated cells (which should be tested by ChIP-seq of Klf4 in NP cells), and thereby initiate stem-cell-specific gene expression patterns during reprogramming (21). For comparison, randomly chosen GGCG sites in mouse genomes are heavily methylated in both ES and NP cells (Figure 2f). Because the bisulphite sequencing method used to generate mouse methylomes in stem cells and NPs (33) does not distinguish between 5mC and 5hmC, or C between 5fC and 5caC (38), the exact modification status of these sites is unknown. All of these sites (Table 2) are located in the gene bodies, as are over half of the Klf4 ChIP-seq sites. While promoter methylation strongly correlates with gene silencing, DNA (hydroxyl)methylation within the gene body is associated with gene activation (39), and the mechanisms of how gene body (hydroxyl)methylation correlates with gene expression are currently under investigation (40). In this respect, it is noteworthy that a very recent study in mouse ES cells (41) indicates that the 5mC dioxygenease Tet1 primarily affects 5hmC levels at gene promoters and transcription start sites, whereas Tet2 mainly modulates those levels in gene bodies.

**Table 2.** Examples of hypomethylation in mouse ES cells and hypermethylation in NP cells within the same 5′-GGCGTG-3′ sequences

RefSeq ID	Gene	Chromosome	CpG start	ES	NP
NM_022312	TnR	chr1	161743113	0	87.5
NM_009271	Src	chr2	157262347	0	100
NM_001127367	DnaJ	chr5	30064128	4.3	87.5
NM_030719	Gatsl2	chr5	134597736	14.3	100
NM_175521	Nyap1	chr5	138173048	0	92
NM_016721	Iqgap1	chr7	87926428	18.2	100
NM_011858	Tenm4	chr7	103642828	18.2	93.3
NM_013875	Pde7b	chr10	20120229	7.7	90
NM_172260	Cep68	chr11	20131937	14.3	100
NM_001039198	Zfhx2	chr14	55690858	0	100
NM_001253759	Enox1	chr14	77918843	6.25	100
NM_134090	Kdelr3	chr15	79349986	12.5	90.9
NR_040470	ncRNA	chr17	34042605	0	100
NM_020625	Zbtb22	chr17	34055672	18.2	100
NM_175276	Fhod3	chr18	25027364	5	86.2

### Structural comparison with Zfp57 and Kaiso

**Figure 3.**
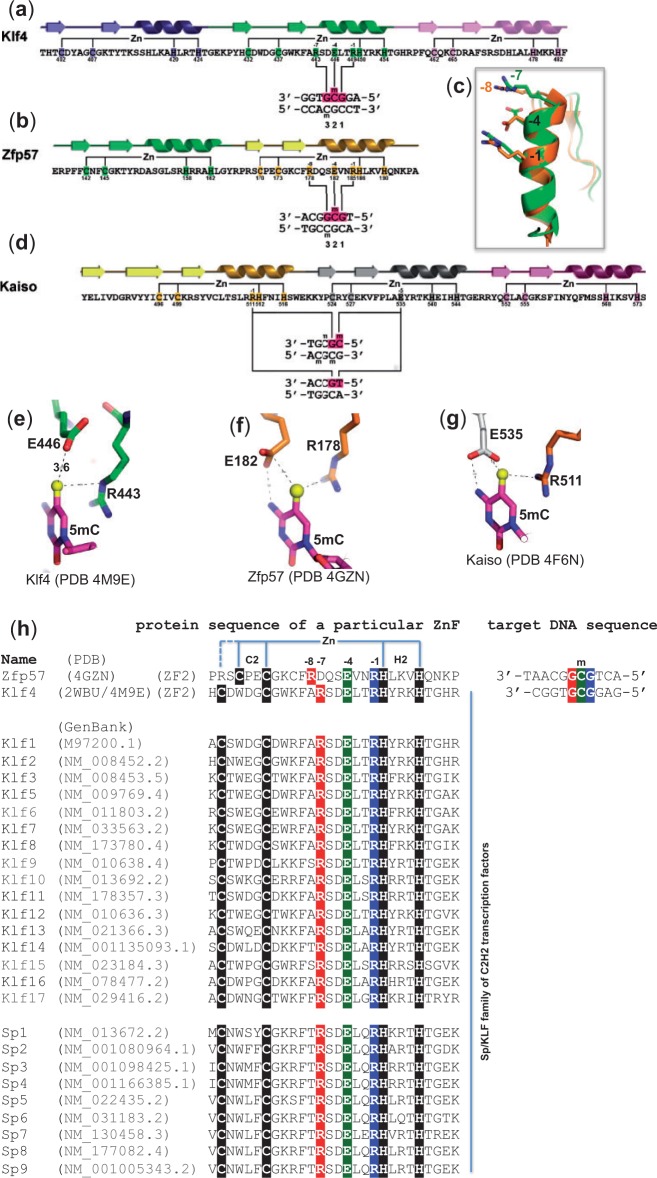
Structural and sequence comparisons of three C2H2 ZnF proteins and their respective DNA interactions. (**a** and **b**) The second ZnF of Klf4 or Zfp57 recognizes a methylated GCG sequence. (**c**) Superimposed GCG-recognition helices of Klf4 (green) and Zfp57 (brown). Arg at −7 position of Klf4 and Arg at −8 position of Zfp57 are spatially aligned. (**d**) Kaiso uses two neighboring ZnF fingers, an arginine at the −1 position of the N-terminal ZnF and a Glu at the −5 position of the C-terminal ZnF, to recognize 5mCpG or TpG. (**e**) In Klf4, together with the Arg at the −7 position, the side chain of Glu at the −4 position forms a C-H…O type of hydrogen bond with the 5mC methyl group. (**f**) In Zfp57, together with the Arg at the -8 position, the side chain of Glu at the -4 position forms a van der Waals contact with the 5mC methyl group and one of its carboxylate oxygen atoms also interacts with the N4 atom of the same 5mC base. (**g**) Although not aligned at the primary sequence level, Kaiso has spatially conserved Arg and Glu, from two neighboring ZnF fingers (d), forming similar interactions with 5mC as that of Zfp57. (**h**) Sequence alignment of the second ZnF of three-ZnF DNA-binding domains among the mouse Sp/Klf family members. The negatively charged glutamate, at the −4 position, appears to be critical in discriminating against the negatively charged carboxylate moiety of 5caC, the final oxidative product of 5mC.

As in previously characterized DNA-binding ZnF structures (42), the DNA base contacts are made by the side chains in the N-terminal portion of the α helix, together with the residue immediately preceding the α helix. Because the first zinc-binding histidine (C_2-4_CX_12_**H**_2-6_H) is located almost always in the middle of the DNA recognition α helix and the spacing between Cys2 and His2 is constant (12 residues), we use the amino acids at positions −1 to −8 (relative to the first zinc-binding histidine) in the following text to discuss the residues making base contact. This numbering scheme allows us to discuss from the perspective of primary sequence without relying on the more variably spaced first position of the α helix. In the ZnF2 of Klf4, the arginine at the −1 position (RH) makes direct base contact to the 5′ Gua, the glutamate at −4 interacts with the central 5mC and the arginine at −7 recognizes the 3′ Gua of methylated GCG (Figure 3a).

Zfp57 recognizes the sequence GMGGCA [where M = 5mC; in the literature, the sequence of the opposite strand TGCCGC was initially used (43)]. Structural analysis of the complex between fully methylated DNA and the tandem two fingers of the mouse Zfp57 DNA-binding domain revealed that the methylated GCG sequence is read by the second ZnF using an arginine at the −1 position (RH), a glutamate at −4 and an arginine at −8 (Figure 3b). Pairwise comparison of the corresponding ZnFs of Klf4 and Zfp57 indicates that the Arg side chains, at the −7 position of Klf4 and −8 in Zfp57, are spatially superimposable (Figure 3c).

Kaiso recognition sequences contain either a methylated CpG (44) or a TpG dinucleotide (45) (both have a methyl group at 5-carbon of the pyrimidine). Structures of the three-ZnF DNA-binding domain of Kaiso, in complex with its methylated CpG or TpG-containing cognate sequences, have been examined (15). Strikingly, interactions similar to those in Klf4 and Zfp57 are observed: an arginine at the −1 position (RH) of ZnF1 interacts with the 3′ guanine of the 5mCpG or TpG dinucleotides, while a glutamate at the −5 position of ZnF2 interacts with 5mC (Figure 3d) or T. The fact that methyl-CpG sites within the consensus recognition sequences of Klf4 and Kaiso can be substituted by TpG raises an intriguing possibility. Perhaps, TpG/CpA sites, which could be methylated by Dnmt3a (24), are selected for when it is advantageous for a particular DNA sequence to be treated as if it is a permanently (hemi)methylated version of the recognition sequence; the shared recognition mechanism for (5mC/T)pG provides that option.

### A noncontiguous Arg–Glu pair for methyl-CpG recognition

The fact that all three ZnF proteins examined here use an arginine and glutamate pair to recognize the methyl group of 5mC (or thymine) implies that glutamate might be a favorable amino acid for recognizing 5mC (or T). However, there is one substantial difference: Glu446 of Klf4 makes a weak C-H…O type of hydrogen bond with the 5mC methyl group (Figure 3e), whereas Glu182 of Zfp57 (like Glu335 of Kaiso) forms a van der Waals contact with the methyl group of 5mC, while one of its carboxylate oxygen atoms also interacts with the N4 atom of the same 5mC base (Figure 3f and g). This difference in interaction might explain the relatively small increase of binding affinity of Klf4 for 5mCpG versus CpG.

The prediction of methyl-CpG binding proteins from primary sequences is still challenging. However, to date, all structurally characterized methyl-CpG binding proteins (except the base-flipping SET and RING finger-associated domain proteins) involve a 5mC-Arg-Gua triad (35). Here we examined structures of three ZnF proteins in complex with modified and unmodified DNA molecules. We propose that the presence of a spatially conserved (nonconsecutive but spatially proximate) Arg–Glu pair in C2H2 ZnF proteins is suggestive of a 5mCpG binding preference (see Figure 3). In the cases of Klf4 and Zfp57, the Arg–Glu pair is within a single ZnF, with an arginine at the −7 or −8 position and a glutamate at −4 (relative to the first zinc-binding histidine). In the case of Kaiso, the methyl-CpG binding residues come from two neighboring ZnF fingers, with an arginine at the −1 position (RH) of the N-terminal ZnF and a Glu at the −5 position of the C-terminal ZnF.

Significantly, sequence identity is >65% among the three-finger DNA-binding domains of the Sp/Klf family (46) (Figure 3h). This conservation implies a similar pattern of DNA recognition among the family members and suggests that other Sp/Klf proteins might be sensitive to DNA methylation status. It is interesting that, while the ubiquitous G/C-rich elements serve as binding sites for all tested Sp/Klf family members, Sp proteins mainly function as transactivators, whereas Klf proteins can activate or repress gene expression (46). The ability to bind either unmethylated or modified elements may contribute to the diverse regulatory mechanisms of Sp/Klf-mediated gene expression via selectively recruiting chromatin cofactors (47). Although our knowledge is currently limited to the genome-wide distribution of 5mC and 5hmC during cellular differentiation, the ability to predict transcription factor sensitivity to DNA modifications is becoming increasingly important.

## ACCESSION NUMBERS

The X-ray structures (coordinates and structure factor files) of Klf4-5mC DNA have been submitted to PDB under accession number 4M9E.

## FUNDING

National Institutes of Health (NIH) [GM049245-20 to X.C.]; The Emory University School of Medicine supported the use of Southeast Regional Collaborative Access Team (SER-CAT) 22-BM beamline at the Advanced Photon Source, Argonne National Laboratory. Use of the Advanced Photon Source was supported by the U.S. Department of Energy, Office of Science; Georgia Research Alliance Eminent Scholar (to X.C.). Funding for open access charge: Waived by Oxford University Press.

*Conflict of interest statement*. None declared.
